# Opium and Belladonna

**Published:** 1867-11

**Authors:** James T. Neuman


### OPIUM AND BELLADONNA.

By James T. Neuman, M.D.

Materia medica has long since taught me that belladona and opium were antagonistic to each other. It has never been my lot, until within a few months past, to test the antagonistic virtues of these powerful medicines.

CASE I. On the 6th of July, 1867, I was called to see a young woman who had taken an ounce of laudanum, as she afterwards confessed, with suicidal intention. I found her insensible, and the pupils contracted. I ordered some strong coffee to be given her. She drank two cupsfull. I had written a prescription for an emetic:—℞. Ipecacuanha, gr. xij, tartarized antimony, gr. ij. M. Upon its arrival, I administered it to her. In a few minutes she vomited copiously. The ejecta were strongly odorized with opium. She still remained in a state of profound stupor. It then occurred to me to try the belladona. I dispatched a man with a prescription which called for one-half ounce of the tincture of belladona, and nine grains of the solid extract. I immediately exhibited a teaspoonful of the tincture: waited twenty minutes and repeated the dose. At the expiration of twenty minutes more, I followed it up with three grains of the solid extract. In the course of two hours, she became sensible, but could not see, and complained of her eyes hurting her. When I left, I told the attendant to give three grains more of the solid extract. The next morning, when I stepped in to see her, the pupils were natural, and sight perfectly restored.

CASE II. A nymph du pave, who had been in the habit of buying morphia, by the bottle, of a druggist just opposite my office; I have, on several occasions, seen her buy sixteen grains, pour it in the palm of her hand, and swallow it with a gusto that would astonish you.

Now, to the case in question. The quantity of morphia that she took, I am unable to determine, but it must have been very great. When I was called in, I found her in a comatose condition; stertorous breathing; small, feeble pulse; pupils contracted; jaws firmly set; extremities cold. Not one drop of liquid could I force down her throat. Here I was in a dilemma. I had every reason to believe that it was morphia she had taken, but what I could do, I could not use the belladonna, because she would not swallow. Must this woman die? *No;* science comes to my aid, in the shape of the hypodermic syringe and atropine, the active principle of the belladonna, in a small space. Now, I have it. I walked over to the store, and ordered the clerk to weigh me out two grains of atropia sulphas, and then add one ounce of water. I went back, took out my syringe, which holds about three drachms, rolled up the patient's sleeve above the elbow, inserted the point beneath the skin, and injected the fluid into the parts. I waited twenty minutes, and applied the syringe to a fresh part. At the expiration of a-half hour, I again used the syringe. By this time, she became somewhat conscious; I then ordered six grains of the solid extract of belladonna to be divided in two parts, one to be given every two hours. She also, like case first, complained of her eyes, and could not discern a single object for about eighteen hours. At the expiration of that time, the pupils assumed their natural appearance, and vision was restored.

Pardon me if I trespass upon your valuable space, as I have still another case to report:

Case III. Mr. B. had in his employ an Irish nurse girl, and to whom he entrusted his child—a bright boy of two years. On the evening of the 4th of September, 1867, she requested leave of absence from him, his wife not being at home. He told her that she might go if she could get the child asleep. Living close by a drug store, she ran off and bought ten cents worth of laudanum, and gave the child a teaspoonful, sat down, and it was soon asleep; and in all human probability, it might have slept forever, had not the father been drawn to his child by some unknown power. He found it breathing very little, and with a pulse scarcely distinguishable.

He sent his man servant after me in great haste. I found the little sufferer's days were numbered, if it was not relieved soon. I ordered it to be stripped, and ice applied to the spine. As soon as I got it, I gave it six drops of the tincture of belladonna every ten minutes for an hour and a-half. At the end of that time, I had the pleasure of hearing the little fellow call for his papa.

Thanks to belladonna! it cheats death of his victim, and causes the rash hand that attempts self-destruction to repent the act.

